# THE ASSOCIATION OF FRAILTY WITH MCI AND DEMENTIA IN A MEMORY DISORDERS CLINIC FOR OLDER VETERANS

**DOI:** 10.1093/geroni/igz038.3253

**Published:** 2019-11-08

**Authors:** Aakashi Shah, Amar Morani, Mercedes Rodriguez-Suarez, Raquel Apracio-Ugarriza, Jorge G Ruiz

**Affiliations:** 1 Department of Public Health, University of Miami Miller School of Medicine, Miami, Florida, United States; 2 Miami VAHS Mental Health Service, Miami, Florida, United States; 3 Miami VAHS Geriatric Research, Education and Clinical Center, Miami, Florida, United States

## Abstract

Frailty, a state of increased vulnerability to stressors due to multiple physiological dysfunction is associated with mild cognitive impairment (MCI) as well as dementia and may moderate its progression. Frailty and cognitive decline are highly prevalent in the Veteran population. The aim of our study was to determine whether frailty is associated with MCI and dementia in older Veterans at a Memory Disorders Clinic. We performed a cross-sectional study of 308 Veterans enrolled in VA Memory Disorders Clinic during 2016-2019. MCI and dementia were diagnosed based on complete clinical assessment including cognitive testing, brain imaging and neuropsychological testing. A 44–item frailty index (FI) was constructed using potential variables (demographics, comorbidities, number of medications, laboratory tests, and activities of daily living). Binomial logistic regression was run using MCI and dementia as outcome variable and frailty status (frail and non-frail) as independent variable. Age, race, marital status, ethnicity, median household income, education, comorbidities, BMI, history of substance abuse, smoking, alcohol, hospitalizations, anticholinergic use, and utilization were considered as covariates. The mean age was 74.43± 8.31 years. 43.2% population was frail (FI>0.21) and 56.8% was non-frail (FI≤0.21). The number of Veterans with MCI and dementia was 114 and 113 respectively. Frailty was significantly and positively associated with dementia (OR: 2.29 95%CI:1.25-4.21, p=0.007) but there was no association with MCI (OR: 1.06 95%CI: 0.605-1.886, p=0.820). Our study results suggest that frailty might not be associated with MCI but has significant association with dementia in a group of Veterans at a Memory Disorder Clinic.

